# A Pilot Phase 2 Randomized Trial to Evaluate the Safety and Potential Efficacy of Etravirine in Friedreich Ataxia Patients

**DOI:** 10.3390/children11080958

**Published:** 2024-08-09

**Authors:** Gabriella Paparella, Cristina Stragà, Nicola Pesenti, Valentina Dal Molin, Gian Antonio Martorel, Vasco Merotto, Cristina Genova, Arianna Piazza, Giuseppe Piccoli, Elena Panzeri, Alessandra Rufini, Roberto Testi, Andrea Martinuzzi

**Affiliations:** 1 Department of Conegliano, Scientific Institute IRCCS E. Medea, 31015 Conegliano, Treviso, Italy; gabriella.paparella@lanostrafamiglia.it (G.P.);; 2 Department of Statistics and Quantitative Methods, Division of Biostatistics, Epidemiology and Public Health, University of Milano-Bicocca, 20126 Milan, Milan, Italy; 3 Department of Bosisio Parini, Scientific Institute IRCCS E. Medea, 23842 Bosisio Parini, Lecco, Italy; 4 Department of Biomedicine and Prevention, University of Rome “Tor Vergata”, 00133 Rome, Italy

**Keywords:** Friedreich ataxia, etravirine, treatment, safety, efficacy

## Abstract

Background: A drug repositioning effort supported the possible use of the anti-HIV drug etravirine as a disease-modifying drug for Friedreich ataxia (FRDA). Etravirine increases frataxin protein and corrects the biochemical defects in cells derived from FRDA patients. Because of these findings, and since etravirine displays a favorable safety profile, we conducted a pilot open-label phase 2 clinical trial assessing the safety and potential efficacy of etravirine in FRDA patients. Methods: Thirty-five patients were stratified into three severity groups and randomized to etravirine 200 mg/day or 400 mg/day. They were treated for 4 months. Safety endpoints were the number and type of adverse events and number of dropouts. Efficacy endpoints were represented by changes in peak oxygen uptake and workload as measured by incremental exercise test, SARA score, cardiac measures, measures of QoL and disability. Data were collected 4 months before the start of the treatment (T − 4), at the start (T0), at the end (T4) and 4 months after the termination of the treatment (T + 4). Results: Etravirine was reasonably tolerated, and adverse events were generally mild. Four months of etravirine treatment did not significantly increase the peak oxygen uptake but was associated with a change in the progression of the SARA score (*p* value < 0.001), compared to the 4 months pre- and post-treatment. It also significantly increased peak workload (*p* value = 0.021). No changes in the cardiac measures were observed. Health and QoL measures showed a worsening at the suspension of the drug. Conclusions: In this open trial etravirine treatment was safe, reasonably well tolerated and appreciably improved neurological function and exercise performance. Even though a placebo effect cannot be ruled out, these results suggest that etravirine may represent a potential therapeutic agent in FRDA deserving testing in a randomized placebo-controlled clinical trial.

## 1. Introduction

### 1.1. Background and Rationale

Friedreich’s ataxia (FRDA) is the most common inherited ataxia, with a prevalence of approximately 1 in 50,000 people affected. It is an autosomal recessive neurodegenerative disorder. The disease is caused by the defective synthesis of frataxin, a mitochondrial protein synthesized by the frataxin gene (*FXN*) [1]. The role of this protein has not yet been fully clarified. We know from the literature that it is a protein responsible for regulating iron–sulfur cluster enzymes within the mitochondria. The defect in frataxin causes a dysfunction of adenosine triphosphate synthesis, accumulation of mitochondrial iron, and increased sensitivity to oxidative stress with a consequent further reduced activity of the mitochondrial respiratory chain. Free radical production and reduced cell respiration lead to cell death, especially in highly energy-dependent tissues such as neurons, cardiomyocytes, and Langerhans pancreatic cells [2,3]. Approximately 96% of patients carry a homozygous intronic GAA expansion that impairs *FXN* gene transcription and translation. The persistence of an intact reading frame within the genome of most FRDA patients opens the way to treatment attempts that target *FXN* gene transcription and messenger RNA (mRNA) translation to increase endogenous frataxin levels and balance the complex metabolic consequences of frataxin deficiency [4]. The age of onset of the disease is typically within the first two decades of life, and wheelchair use is necessary ten to fifteen years after disease onset [5]. The disease is characterized by a relentless progression involving motor coordination (ataxia), heart function and structure (hypertrophic cardiomyopathy), exercise capacity (impaired bioenergetics with reduced aerobic capacity), endocrine function (diabetes), and visual and auditory function (optic atrophy and hearing loss) [5,6,7].

Recently, it has been shown that the drug etravirine (ETR), a low-molecular-weight agent currently used in human immunodeficiency virus (HIV) therapy with an excellent safety profile and tolerability, stimulates the translation of the *FXN* gene and promotes cellular accumulation of frataxin. It has been suggested that ETR might increase frataxin protein levels by enhancing the translation efficiency of frataxin mRNA [8]. The mechanism proposed is based on experimental evidence showing that, in patient-derived frataxin-deficient cells, exposure to ETR induces the redistribution of frataxin mRNA onto transcriptionally active polysomes, thus specifically enhancing frataxin mRNA transcription rates. Transcription efficiency of other mRNAs is not affected by ETR. The mechanism of action that leads to increased frataxin protein levels is therefore different from the mechanism that enables ETR to inhibit the HIV reverse transcriptase.

By screening a library of 853 Food and Drug Administration (FDA)-approved drugs, few drugs were identified capable of upregulating frataxin in vitro. Among them, ETR was selected for its generally favorable safety profile and its significant effect on frataxin levels. Preclinical studies in cells derived from FRDA patients have confirmed a role of ETR in promoting the accumulation of frataxin, correcting the biochemical damage caused by frataxin deficiency and protecting cells from oxidative damage. ETR induces an overexpression of the frataxin protein by a mechanism of action that presumably involves the redistribution of the frataxin mRNA onto polyribosomes and consequently improved efficiency of protein translation [8]. In addition, ETR is able to upregulate the frataxin protein in the heart and skeletal muscle of frataxin-defective YG8 mice after oral administration for 5 days (unpublished). These results assume even greater significance considering that reconstitution of frataxin levels in vitro was obtained with ETR concentrations corresponding to those achieved in plasma during anti-HIV ETR therapy at standard doses (200–400 mg/day). 

ETR is a diarylpyrimidine that acts as a non-nucleoside reverse transcriptase inhibitor. It was approved in 2008 by the FDA (trade name Intelence) for the treatment of patients affected by HIV that are resistant to other drugs. In 2018, the label was extended to include patients 2 years of age and older. The FDA label reports no contraindications. ETR is administered orally in tablets.

Treatment options are currently very limited for FRDA patients, with omaveloxolone, or Omav, being the first and only drug approved in 2023 by the FDA for adult and adolescent FRDA patients 16 years of age and older, as capable of improving mFARS score and slowing disease progression after chronic treatment. Omav is a nuclear erythroid-derived 2-related factor 2 (Nrf2) activator. In FRDA, there is reduced activation of Nrf2 in cells. Nrf2 is a transcription factor essential for counteracting oxidative stress and the consequent cellular damage, reducing the expression of the inflammatory cytokines TNF-alpha and IL-6 [9,10].

### 1.2. Objectives

Because of its ability to upregulate frataxin protein and rescue phenotypic defects in FRDA patient-derived cells and because of its safety and approved use in children, ETR represents an attractive potential disease-modifying drug for FRDA. Our goal is to test this drug in patients with FRDA through a pilot open phase 2 clinical study. The study aims to explore the transferability from laboratory to clinical practice of the encouraging results obtained on human cells and animal models and to offer ground for further placebo-controlled trials. The questions that the study intends to address are the following:

1. Will ETR in FRDA patients continue to maintain the same safety and tolerability profile shown so far in licensed clinical use?
2. Will in vivo ETR in FRDA patients with the doses proposed in this study, capable of ensuring serum concentrations similar to those tested in vitro, be associated with significant variation of the indicators used as efficacy endpoints?

## 2. Materials and Methods

### 2.1. Study Design 

We designed a monocenter randomized pilot open-label phase 2 clinical trial to evaluate the safety and potential efficacy of ETR in FRDA at two doses. The patients were stratified by severity and assigned randomly to two different groups treated with different dosages (200 mg/day or 400 mg/day). The 400 mg/day dose was chosen as this is the full dose recommended in the current label. The dose of 200 mg/day was chosen based on feedback from many patients with FRDA who are already using ETR off-label.

The study includes 5 visits comprising a period of treatment of 4 months, preceded by 4 months and followed by an additional 4 months of study in absence of treatment to account for intra-individual disease progression.

This design and this treatment time were chosen on the basis of the experience gained in the study “Safety and efficacy of treatment with γIFN in subjects with FRDA” recently concluded by the promoter, in which the comparison between disease progression in the absence of treatment and in the presence of treatment provided significant data and in which this variation was already found in the third month of treatment [11].

### 2.2. Setting

The study was performed at the “Eugenio Medea’’ Scientific Institute in Conegliano (Treviso, Italy) from September 2020 to January 2023, with recruitment concluded by January 2022.

### 2.3. Participants

Recruitment was performed by advertising the study through the patients’ associations and among FRDA patients currently followed at the site.

Of the 38 patients assessed for eligibility, 35 subjects were enrolled after verification of their willingness to participate in the study and compliance with the inclusion criteria.

Patients were required to have molecularly defined FRDA diagnosis (at least one expanded *FXN* allele) and be ≥10 years of age and ≤40 years of age. They also had to be able to complete an incremental exercise test defined by the ability to ride a cycle ergometer with upper limbs at least 45 rpm with no added resistance for 3 min. Moreover, they had to weigh > 30 kg.

Patients were excluded if they had HIV, HBV or HCV infection, severe organ failure, history of clinically significant cardiac disease and uncontrolled diabetes. Another exclusion criteria was known hypersensitivity to ETR or to any component of the study drug. Patients were required to discontinue other experimental therapeutics for FRDA at least 4 weeks before study entry. No participation in other experimental studies was allowed. Patients were then asked to maintain their typical exercise routine.

All participants and their parents/legal tutors were informed regarding the experimental nature of the study and signed the informed consent in accordance with the Declaration of Helsinki (World Medical Association, 1964). The study has been reviewed and approved by the competent Ethics Committee (Prot. No. 0103439, 1 July 2020). This study was registered at www.clinicaltrial.gov (NCT04273165).

### 2.4. Interventions

Patients received a dose of 200 mg/day (1 tablet/day in the morning) or 400 mg/day (2 tablets/day morning and evening) for 4 months.

The first dose was given under direct medical supervision in the facility. The subsequent treatment was carried on at home following the prescription given to the patients.

### 2.5. Study Endpoints

The primary endpoint was the safety and tolerability of ETR at 200 or 400 mg daily dosage in FRDA subjects aged 10–40 years. To monitor it, the patients were subjected to physical examinations including vital parameter assessment, skin inspection, blood tests (clinical chemistry, hematology), and urinalysis. These assessments were performed 4 months before the start of the treatment (T − 4), at the start of the treatment (T0), after 2 months (T2), at the end of the treatment (T4), and 4 months after the termination of the treatment (T + 4). In addition, a checklist listing all known adverse events (AEs) associated with ETR was distributed to all patients and reviewed at each follow-up visit. Any additional AE was registered irrespective of its likely connection with the treatment.

The secondary endpoint was the assessment of the efficacy of the treatment. The main outcome was peak oxygen uptake (peak VO_2_) attained during the incremental exercise test. Other outcomes were:

- peak workload (peak W);
- neurological progression measured with Scale for the Assessment and Rating of Ataxia (SARA) [12];
- echocardiography (septal wall thickness) and electrocardiogram (Sokolow–Lyon index) parameters;
- frataxin protein levels in peripheral blood mononuclear cells and molecular analysis of frataxin mRNA translation efficiency;
- quality of life evaluated with Short Form Health Survey 36 (SF-36) [13];
- disability, assessed by World Health Organization Disability Assessment Schedule 2.0 (WHODAS 2.0) 36-item self-administered version [14].

These measures were assessed at T − 4, T0, T4, and T + 4, while the SARA scale was also assessed at T2. 

**Figure 1:**
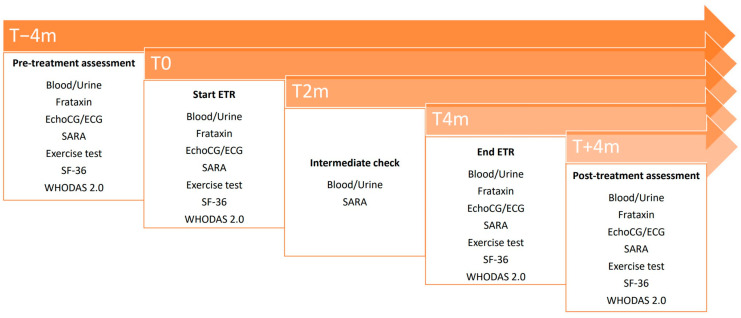
Study scheme. Timeline of the study.

The study scheme is represented in Figure 1.

#### 2.5.1. Incremental Exercise Test

It has been shown that peak VO_2_ normalized by weight is a very stable objective measure of aerobic capacity [15,16]. FRDA is associated with a significant decrease (about 50% less) in aerobic fitness as measured by standardized exercise testing, and its measure has been employed as an endpoint in FRDA intervention trials several times [9,17,18,19]. The incremental exercise test was performed on a cycle ergometer for upper limbs (Cosmed arm ergometer 400 P), and the assessments included peak VO_2_ (in mL/min/kg) and peak W (in Watt). 

The test included a 1 min warm-up period at 0 W workload, maintaining a cadence of at least 45 rpm, followed by a 2 W increase in work rate every one minute. Subjects were encouraged to work to exhaustion. This mild exercise protocol was chosen to include patients with a wide range of severity (SARA score).

#### 2.5.2. Neurological Measure

The SARA scale was used as the neurological measure. It is based on a semi-quantitative clinical assessment of cerebellar ataxia (spinocerebellar, Friedreich’s, and sporadic ataxia) on an impairment level. SARA has 8 items (gait, stance, sitting, speech, finger-chase, nose-finger, fast alternating movements, heel-shin). It has a maximum score of 40 (severe ataxia) and a minimum of 0 (no ataxia).

#### 2.5.3. Cardiac Parameters

The heart is primarily affected in FRDA, showing progressive cardiac hypertrophy. Cardiomyopathy is the main determinant of survival in this population. The electrocardiogram (ECG) changes summarized by the Sokolow–Lyon index (∆%QRS-voltage) are reliable indicators of ventricular hypertrophy. Echocardiography (EchoCG) can detect and measure very precisely the extent and severity of heart hypertrophic changes. We measured interventricular septal wall thickness (in mm) and Sokolow–Lyon index (in mm).

#### 2.5.4. Health and Disability Measures

SF-36 is a self-administered questionnaire, completed by the patient, which aims to quantify the state of health and measure the health-related quality of life. The questionnaire explores 8 domains that are scored separately:

- Physical functioning
- Physical role limitations
- Bodily pain
- General health
- Vitality
- Social functioning
- Emotional role limitations
- Mental health.

The scores are transformed to range from 0 (worst possible health) to 100 (best possible health).

WHODAS 2.0 is an internationally validated, widely applied, disease non-specific measure of disability developed by WHO within the framework of the biopsychosocial model of functioning and disability. It asks the subject to score in terms of severity and duration any difficulty experienced in the 30 previous days in 6 main life areas:

- Cognition—understanding and communicating;
- Mobility—moving and getting around;
- Self-care—hygiene, dressing, eating and staying alone;
- Getting along—interacting with other people;
- Life activities—domestic responsibilities, leisure, work and school;
- Participation—joining in community activities.

The self-administered version was used. Domain-specific scores and a summary score are calculated, and the results were computed as normalized scores that range from 0 (no disability) to 100 (full disability).

These measures were administered only to subjects ≥16 years of age.

### 2.6. Sample Size Calculation

Sample size was calculated considering the principal efficacy outcome. The VO_2_max test on the cycle ergometer in normal subjects shows a value > 16.2 mL/min/kg with a test–retest variability of ±4 mL/min/Kg. This parameter shows values reduced by about 50% in patients with FRDA. Assuming an expected variation of the range of 0–2% between time points T − 4 and T0 and of +20% between T0 and T4 (α error of 0.05, statistical power of 80%), the required number of participants was 30. Assuming approximately 15% of the trial dropped out due to non-safety issues (e.g., failure to complete assessments, non-compliance with drug regimen, or withdrawal of consent), the enrollment goal was set at 35 patients.

### 2.7. Randomization

After being stratified into 3 severity groups defined by the SARA score obtained at T − 4 (<20, 20–30, >30), the recruited subjects were assigned to the dose of 200 mg/day or 400 mg/day randomly by computer-generated sequencing, with a 1:1 allocation ratio using blocks of 4 within each group to ensure that the intervention was balanced within each stratum.

### 2.8. Statistical Analysis

Descriptive statistics were obtained for the population characteristics. Continuous variables were described as mean (SD) or median (IQr), while categorical variables were expressed as number (percentage). The comparison between the two dose groups was performed using *t*-test or Mann–Whitney U test for continuous variables and Fisher’s exact test for categorical variables. The effect of the treatment on the considered outcomes was studied using linear mixed-effect models with subject as random effect. Post hoc tests were performed for the significant models’ effects. All models were adjusted for age, severity, and dosage. Finally, multiple linear models were used to study the effect of dosage and severity (independent variables) on differences in outcomes (dependent variables) during the treatment period (between T0 and T4) and in the post-treatment period (between T4 and T + 4), adjusting for age. 

Values of *p* < 0.05 were considered statistically significant. Statistical analyses were performed using R version 4.4.0 [20].

## 3. Results

### 3.1. Participant Flow

**Figure 2:**
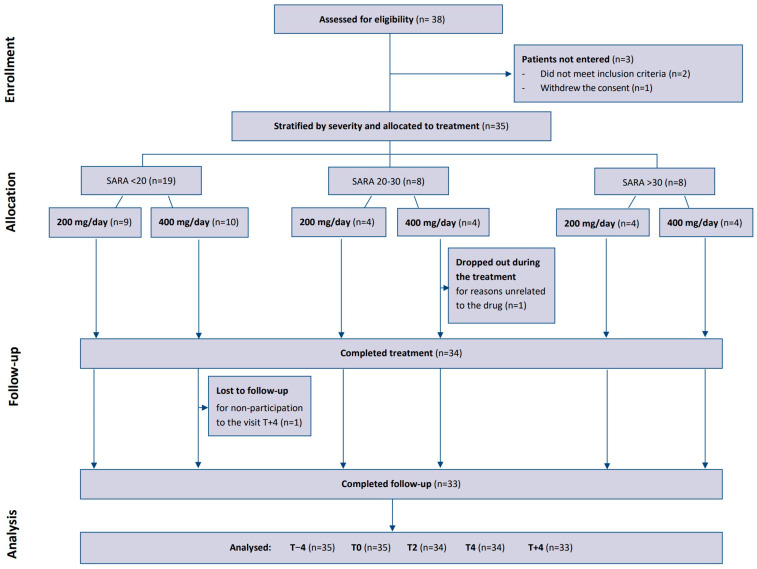
CONSORT flowchart of the study.

With the goal of enrolling 35 participants, 38 patients were screened. Two of them were excluded because they did not meet the criteria of inclusion and one withdrew consent. The enrolled patients were divided into 3 severity classes: 19 with SARA < 20, 8 with SARA 20–30, and 8 with SARA > 30. In the first group, 9 patients were randomized to ETR at doses of 200 mg/day, and 10 patients were randomized at doses of 400 mg/day. In the other two groups, 4 patients received 200 mg and 4 assumed 400 mg. One participant withdrew after completing the T4 visit. Another patient dropped out during the treatment for reasons unrelated to the drug (see Figure 2).

### 3.2. Patient Features

The patients are 30 adults and 5 children, 19 females and 16 males. The average age was 25.0 (7.1) (mean (SD)) years and disease duration of 13.2 (6.0) years, with an age at onset of 11.8 (5.7) years. The mean overall SARA score at T − 4 was 20.7 (8.2), while for the first group of severity it was 14.4 (3.6), for the second one 24.8 (2.6), and the third one 32.2 (1.1). Thirty-three patients were homozygous for GAA repeat expansion (defined as being above 66 repeats) and 2 had a repeat expansion on one allele and a point mutation on the other. For 5 patients, the precise number of triplets was not available, while for the others the mean GAA repeat expansion in the short allele (GAAsr) was 665.5 (189.0) while in the long allele (GAAlr) 862.9 (203.9).

Patient demographics and clinical data are presented in Table 1.

### 3.3. Safety

One event of symptomatic supraventricular tachycardia (SAE), judged unrelated to study drug, occurred. There were no treatment interruptions or discontinuations due to tolerability issues.

Overall, AEs were generally mild in severity and were consistent with the Summary of Product Characteristics.

Table 2 shows the AEs reported by the patients or detected in the physical examination, dividing them into the first two months and the last two months of treatment. The most commonly reported AE was headache (47% of subjects in the first period of treatment), followed by diarrhea (21%), nausea (12%), skin rash (12%), fatigue (9%), and hypotension (6%). In particular, it should be noted that the most frequent AEs appeared and resolved in the first two weeks of treatment, with the exception of 4 cases that presented sporadic episodes of headaches, diarrhea, and nausea even in the last two months, but also after the suspension in the follow-up period. Most events resolved spontaneously, except for 2 cases of diarrhea and 7 cases of headache, in which targeted drug therapy was taken, respectively, lactic acid ferments and paracetamol or other non-steroidal anti-inflammatory drugs.

Table 3 shows the main alterations detected in blood and urine tests that occurred while taking the drug. Five patients had a lipid alteration, in particular 2 patients had an increase of total cholesterol, 1 of triglycerides, and 2 of both. There was 1 case of liver enzymes transient elevation (AST/ALT), 1 case of amylase rise, and 1 case of mild leukopenia. There were also 2 cases (6%) of slight increase in glycated hemoglobin, but within the limits of 10%. These values normalized when the treatment was stopped.

These effects were not dose-dependent. They were observed in both high- and low-dose treatment.

### 3.4. Efficacy 

#### 3.4.1. Variations in Clinical and Functional Outcomes

**Figure 3:**
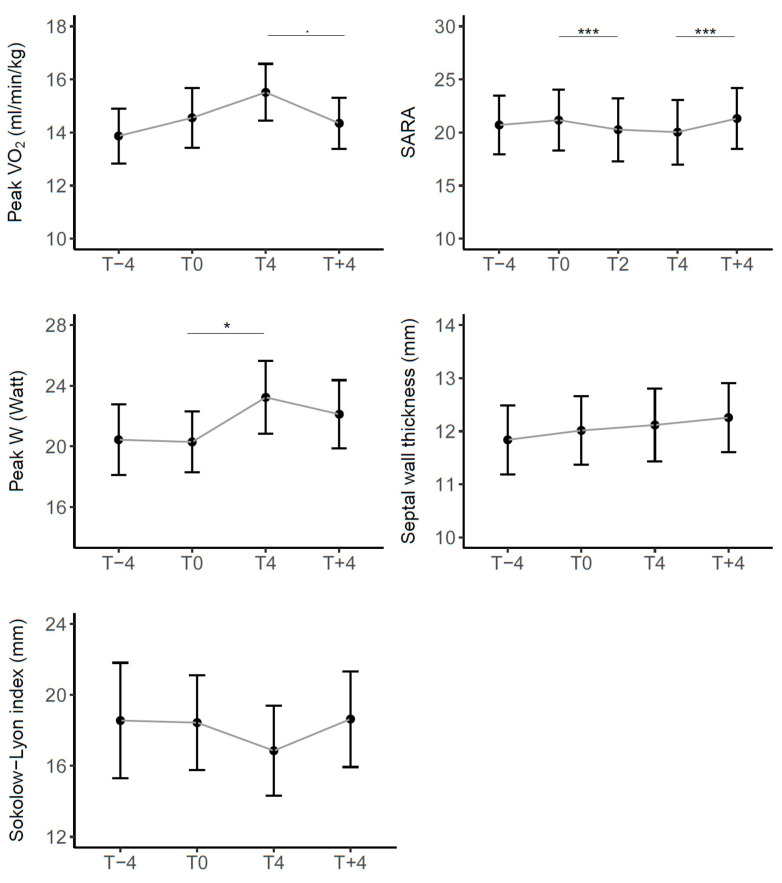
Trend of clinical and functional outcomes over time in the entire cohort of patients. Sig: *** < 0.001, * < 0.05, . < 0.1.

Regarding clinical and functional outcomes, Figure 3 represents the trend of the mean scores in all patients in timepoints (scores in Table S1). Except for the septal wall thickness, the variations were in the direction of a functional improvement taking the drug (T4 vs. T0) that reversed at drug discontinuation (T + 4 vs. T4).

In particular, from mixed effect models results, described in Table 4, peak VO_2_ showed an average increase, although not statistically significant, with the drug of 0.959 mL/min/kg, while the decrease with the suspension (−1.145 mL/min/kg) approached statistical significance (*p* value = 0.088). We observed a slight improvement from T − 4 to T0, which can be considered physiological or a consequence of familiarization with the test.

Among the secondary endpoints, it should be noted that the SARA scale documented the expected progression of the disease from T − 4 to T0 with a mean worsening of 0.456 points, while a significant improvement with the treatment (from 21.16 to 20.03, mean reduction score −1.132, *p* value < 0.001). There was also a significant worsening when the drug was discontinued (from 20.03 to 21.32, average increase score 1.229, *p* value < 0.001). 

For peak W there was a significant improvement while on drug treatment (from 20.29 to 23.24, average increase 2.941 Watt, *p* value = 0.021) and a subsequent worsening with a non-significant decrease with the suspension (−1.037 Watt).

Regarding cardiac measures, the Sokolow–Lyon index showed a similar trend to that observed for SARA, while the septal wall thickness displayed a slight but constant worsening trend during the entire duration of the study, although in both cases there were no significant differences between timepoints.

Considering the mean differences by severity and dose (Table 5), we found the same trend in all three severity groups and for both doses, that is, an improvement in the scores during treatment and a worsening at suspension, except for the septal wall thickness. 

Specifically, on the SARA scale, at lower severity there appears to be a greater average improving effect of the drug, but also a greater worsening effect upon discontinuation. Regarding peak VO_2_ and Sokolow–Lyon index, no particular trends are noted for different severities, while for peak W the effect appears greater in more severe patients.

These results are supported by the regression model results (Table 6). We observed a significant difference for the SARA score between the high severity and low severity groups both during treatment (mean difference of 1.398 points, *p* value = 0.010) and after treatment (mean difference of −1.552 points, *p* value = 0.002). In contrast, for peak VO_2_ and peak W, no significant differences were observed between the severity groups. 

Regarding dosage, the observed mean effects appeared more pronounced for high dosages, both in terms of improvement during the treatment and in terms of worsening at suspension, but not significant. Also for cardiac parameters, no significant differences were observed between severity and dosage groups.

#### 3.4.2. Variations in SF-36 and WHODAS 2.0 Questionnaires

**Figure 4:**
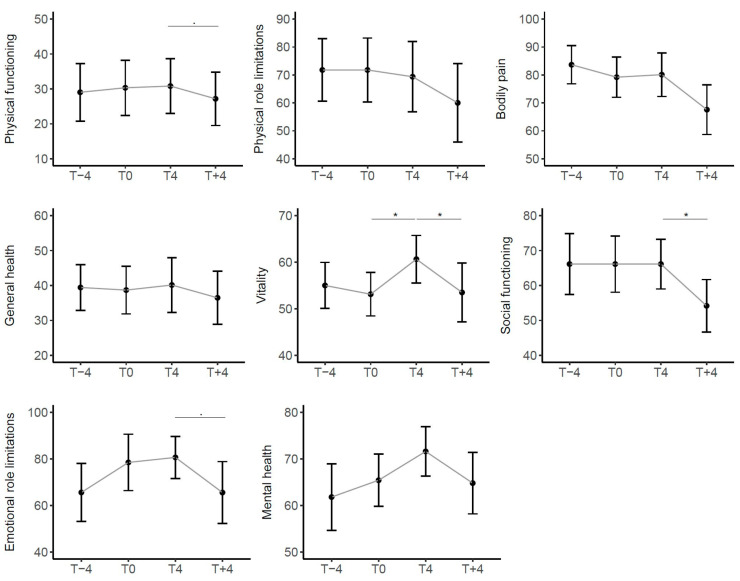
Trend of scores in SF-36 over time in the entire cohort of patients. Scores range from 0 (worst possible health) to 100 (best possible health). Sig: * < 0.05, . < 0.1.

In all eight domains of the SF-36, the average score was fairly stable before the start of treatment, with the exception of scores for Emotional Role limitations and Mental Health domains, where there was a non-significant improvement associated with enrollment in the study that persisted during treatment. The domain that improved significantly during the treatment was Vitality, which then worsened significantly upon suspending the drug, together with the domain of Social Functioning. The trend of all domains in the four months following the end of treatment indicated a worsening (Figure 4).

**Figure 5:**
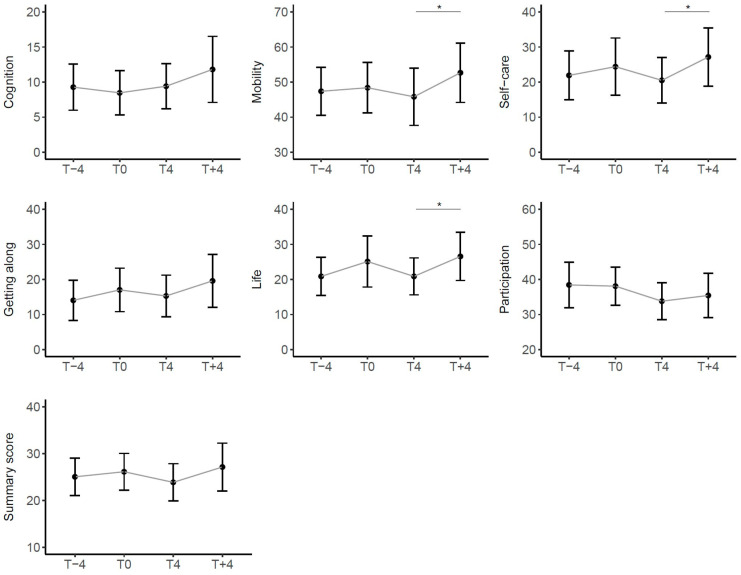
Trend of scores in WHODAS 2.0 over time in the entire cohort of patients. Scores range from 0 (no disability) to 100 (full disability). Sig: * < 0.05.

We then considered the trend of the WHODAS 2.0 summary score and its six domains. With the exception of the Cognition domain that remained stable, these scores also showed a trend of worsening in the 4 months before taking the drug, of improvement during treatment and of subsequent worsening after withdrawal. In particular, the latter was statistically significant in the Mobility, Self-care, and Life Activities domains. (Figure 5).

#### 3.4.3. Variations in Frataxin

The sample for the analysis of the frataxin protein and mRNA was shipped to the laboratory identified for the extraction and preparation of the material and then to the laboratory identified for the analysis. The shipments were to be completed within strict time frames but the logistics complications linked to the concurrent pandemic condition caused fatal deterioration of the samples, preventing any reliable analytical result.

## 4. Discussion

We report here the results of a pilot open-label clinical trial testing ETR in a group of 35 FRDA patients. This is the first study to evaluate the effect of ETR in patients with FRDA, and it demonstrates that ETR represents a relatively safe drug with a potential role in the treatment of FRDA.

The choice of drug was driven by the pre-clinical research results, reinforcing the strength of the translational potential of the drug repurposing research, particularly in the context of rare diseases such as FRDA [21,22]. The design was inspired by the goal of appreciating the capacity of the tested drug to modify the disease’s natural history, while the duration was chosen to meet the preliminary results obtained on the animal model and to keep the trial duration at the minimum, maximizing efficiency. Both criteria, albeit convenient, could have also amplified the confounding placebo effect that typically lasts a few months and affects clinical scale results.

This phase 2 study was aimed primarily at addressing the safety and tolerability of ETR in FRDA patients. We found only minor adverse effects, both qualitatively and quantitatively similar to what had already been observed in other studies on ETR for other diseases [23], which indicates that ETR is generally safe and reasonably tolerated by FRDA patients. The single SAE, albeit deemed not related to treatment, calls for special attention to cardiac parameters in FRDA subjects taking ETR.

In addition to the overall safety of ETR in FRDA, we evaluated efficacy. 

This protocol, which provides for a total duration of one year (4 months pre-treatment, 4 months with treatment, and 4 months post-treatment), made it possible to evaluate the patient in a year of disease and how ETR affects the disease trend. We document the expected progression of the disease in the pre- and post-treatment period, from T − 4 to T0 and from T4 to T + 4. The extension of the study to 4 months before treatment start and 4 months after treatment termination gives the possibility to study and compare, for each patient, the disease progression in the absence of therapy and the disease course after termination of therapy. In this design, each subject is an effective control for him/herself. The chosen main secondary endpoint (peak VO_2_) was not met, even though an absolute mean increase was observed during treatment that dropped after treatment termination. The additional secondary outcomes SARA score and Wmax instead showed a positive change during treatment. From T0 to T4, SARA score increase was stopped and even reversed. This study was able to record a remarkable average decrease of about 1.1 points in the SARA scale, after 4 months of treatment. This improvement is significant, given that 1 SARA point would be the expected disease progression in one year [24]. In addition, at the incremental exercise test, there was a significant increase in peak W. 

Given the open nature of the trial we cannot exclude a placebo effect, that in other studies was recorded in similar timing and magnitude [9]. Nevertheless, the significance of the observed SARA and workload variations supports the need for a longer placebo-controlled trial.

The behavior of the cardiac parameters is probably strongly influenced by the short duration of the study. This is especially true for the morphologic changes in septal wall. In contrast to the neurological outcomes, longitudinal studies detailing the progression of the cardiac disease are sparse and not adequate to provide an expected timing for the linear progression of disease, making it difficult to determine the rate of change in cardiac parameters. The thickening of the interventricular septum is one of the most common manifestations in patients with Friedreich’s ataxia, and the expansion of GAA repetitions can affect the speed and extent of this thickening; thus, in our cohort of heterogeneous GAA size, it becomes difficult to expect a similar rate of progression for the whole group.

In the SF-36 questionnaire on quality of life, there was a variability of trends in different domains. The constant was a worsening trend in all of them at the suspension of the drug. The vitality, intended as energy and lack of fatigue, followed the trend seen earlier, namely an initial worsening, a significant improvement with the drug, and a significant decline after suspension. As for the emotional and mental health aspects, it should be noted that there was an improvement starting from enrollment in the study, and this trend could signal a placebo effect linked to the expectation of healing related to trial participation.

The WHODAS 2.0 questionnaire on the impact of disability showed a non-significant improvement during the treatment in all domains, except for the domain of cognition, in which we did not expect to find it, given that the questions consider aspects that are not part of the core impairment in ataxic patients. Also in this questionnaire, there was a worsening after discontinuation of the drug in all domains, that was especially significant for the domains of daily life and mobility. 

As these are self-administered questionnaires, we cannot exclude that the improvement observed during the administration of the drug in most domains as well as the worsening at suspension in all of them are the result of a placebo/nocebo effect related to psychological impact of being part of a treatment trial.

However, the trend of these PROMs is the same as the other efficacy indicators, which are objective clinical parameters and not self-administered questionnaires.

In support of these findings, patients and caregivers reported additional notations not captured by any formal tool: reduced fatigue and increased energy and endurance; less help in moving and transferring, greater stability with reduction of falls; improved manual skills and writing; reduced dysphagia episodes, improved swallowing; improved mood; improved voice quality and speech; reduced urinary urgency. These informal reports may guide the development of more tailored PROMs. The perceived efficacy was such that 22 patients requested to start an off-label treatment with ETR.

We originally planned to also quantitate the amount of frataxin mRNA and protein in peripheral blood cells, as an additional efficacy measure. However, the limited amount of blood that could be drawn from most patients, combined with sample shipment issues due to the pandemic, did not allow a consistent extraction of frataxin mRNA and protein from all samples, preventing a complete and reliable analysis. In future trials, the attempt to study frataxin protein or mRNA may consider targeting tissues with higher frataxin content such as cells in urinary sediments or skeletal muscle obtained by needle biopsy.

Patients of all stages of the disease were enrolled in our study. The study cohort consisted of a heterogeneous patient population, including ambulatory patients at disease onset and patients in more advanced clinical stages who had already been in a wheelchair for years. Our study shows that there is a greater response to ETR in subjects with mild severity, while in severely affected subjects with SARA > 30, the effect is smaller. This result could be explained by the ceiling effect of the scale. We observed a ceiling effect in particular for the items concerning gait, sitting position, and heel-shin slip. The ceiling effect of SARA has already been observed for patients with FRDA by several authors [24,25]. The ceiling effect of SARA may have underestimated the clinical variations in severely affected patients.

Currently, Omav is the only drug approved for FRDA. Possible use of ETR in combination, as already suggested [26] could be considered, since its mechanism differs from Omav and also from other proposed or tested treatments (γIFN, Vatiquinone, Elamipretide, Leriglitazone).

### Limitations of the Study

A major limitation of this study, as already stated, is the absence of a placebo group.

Although the number of participants in the study is relevant considering that FRDA is a rare disease, the cohort was too small to be stratified according to expansion size and disease duration. Moreover, the chosen duration of the trial, while convenient in terms of sustainability, reduces the opportunity to detect medium-term effects of ETR treatment. The unavailability of the frataxin and frataxin mRNA measures weakens the study relevance, preventing the demonstration of the biological changes associated with treatment.

## 5. Conclusions

In this study, regarding the safety of etravirine in FRDA patients, there was no treatment-related drop-out and good tolerability even at full dosage (400 mg/day). The significant variations in efficacy indicators do not allow conclusions to be drawn in terms of treatment effectiveness given the short-term administration of the drug and the short duration and pilot-open nature of the study. This does not allow a differentiation of actual effects from a placebo effect, but the results represent a promising basis for the design of a larger and longer placebo-controlled trial. For a future trial, the higher dose may be advised since it was safe and tolerated as the lower dose and marginally associated with better results.

## Figures and Tables

**Table 1:** Demographic and clinical data of patients included in this study and comparisons between dose groups.

	Overall (N = 35)	Dose 200 (N = 17)	Dose 400 (N = 18)	*p* Value
Patient type, N (%)				>0.999
Adult	30 (85.7)	15 (88.2)	15 (83.3)	
Children	5 (14.3)	2 (11.8)	3 (16.7)	
Male, N (%)	16 (45.7)	5 (29.4)	11 (61.1)	0.123
Severity, N (%)				0.988
SARA < 20	19 (54.3)	9 (52.9)	10 (55.6)	
SARA 20–30	8 (22.9)	4 (23.5)	4 (22.2)	
SARA > 30	8 (22.9)	4 (23.5)	4 (22.2)	
GAA sr	665.50 (189.02)	670.87 (197.10)	660.13 (187.35)	0.880
GAA lr	862.89 (203.86)	917.64 (226.81)	808.14 (168.56)	0.159
Age at onset (years), mean (SD)	11.83 (5.70)	10.65 (4.20)	12.94 (6.76)	0.239
Age (years), mean (SD)	25.00 (7.14)	23.53 (5.01)	26.39 (8.61)	0.242
Disease duration (years), mean (SD)	13.17 (6.00)	12.88 (5.29)	13.44 (6.75)	0.786
Weight (kg), mean (SD)	63.07 (14.11)	62.82 (15.94)	63.31 (12.60)	0.921
Years of education, median [IQR]	13.00 [13.00, 16.50]	14.00 [13.00, 17.00]	13.00 [13.00, 15.50]	0.458

*p* values were obtained using *t* test, Mann–Whitney U Test, or Fisher’s exact test. GAAsr, GAA short repeat; GAAlr, GAA long repeat.

**Table 2:** Number (%) of subjects with AEs detected during the study.

	Months 1 and 2	Months 3 and 4
Headache	16 (47%)	3 (9%)
Diarrhea	7 (21%)	1 (3%)
Nausea	4 (12%)	1 (3%)
Skin Rash	4 (12%)	0
Other:		
Fatigue	3 (9%)	0
Hypotension	2 (6%)	0
Tachycardia	1 (3%)	1 (3%)
Vomit	1 (3%)	1 (3%)
Constipation	1 (3%)	0
Blurred vision	1 (3%)	0
Sweating	1 (3%)	0
Urinary urgency	1 (3%)	0
Calf cramps	1 (3%)	0

**Table 3:** Number (%) of alterations in blood tests during treatment. + indicates an increase and − a decrease.

	N (%)
Total cholesterol +	4 (12%)
Triglycerides +	3 (9%)
AST/ALT +	1 (3%)
Amylase +	1 (3%)
White blood cells −	1 (3%)

**Table 4:** Mixed models’ post hoc differences for clinical and functional outcomes between timepoints.

	T0 vs. T − 4	T2 vs. T0	T4 vs. T2	T4 vs. T0	T + 4 vs. T4
	Start ETR vs. Pre-treatment	Intermediate check vs. Start ETR	End ETR vs. Intermediate check	End ETR vs. Start ETR	Post-treatment vs. End ETR
Peak VO_2_	0.685 (0.619)			0.959 (0.224)	−1.145 (0.088)
SARA	0.456 (0.243)	−0.897 (<0.001)	−0.235 (0.914)	−1.132 (<0.001)	1.229 (<0.001)
Peak W	−0.147 (>0.999)			2.941 (0.021)	−1.037 (0.905)
Septal wall thickness	0.176			0.103	0.111
Sokolow-Lyon index	−0.118			−1.582	1.808

Estimated difference between times (*p* values). *p* values for post hoc are shown only for outcomes with significant effect at mixed model (peak VO_2_, SARA and peak W).

**Table 5:** Mean (SD) differences in scores during treatment and after suspension by severity and dosage.

	SARA < 20 (N = 19)	SARA 20–30 (N = 7)	SARA > 30 (N = 8)	Dose 200 (N = 17)	Dose 400 (N = 17)
Peak VO_2_					
delta T4–T0	0.51 (2.26)	1.81 (1.88)	1.29 (2.45)	0.80 (2.80)	1.12 (1.55)
delta T + 4–T4	−1.42 (2.84)	−1.21 (2.15)	−0.36 (1.18)	−0.99 (2.60)	−1.25 (2.19)
SARA					
delta T4–T0	−1.53 (1.03)	−1.21 (1.82)	−0.12 (0.35)	−0.97 (1.21)	−1.29 (1.30)
delta T + 4–T4	1.78 (1.23)	1.14 (1.14)	0.06 (0.50)	0.94 (1.14)	1.53 (1.36)
Peak W					
delta T4–T0	2.95 (4.34)	1.43 (2.76)	4.25 (8.45)	2.71 (3.24)	3.18 (6.82)
delta T + 4–T4	−1.00 (2.77)	1.43 (1.90)	−3.00 (11.36)	−0.71 (2.91)	−1.25 (8.13)
Septal wall thickness					
delta T4–T0	0.11 (0.86)	0.43 (0.84)	−0.19 (1.07)	0.06 (0.58)	0.15 (1.16)
delta T + 4–T4	0.19 (0.86)	0.07 (0.61)	0.06 (0.68)	−0.09 (0.73)	0.38 (0.72)
Sokolow-Lyon index					
delta T4–T0	−1.31 (3.64)	−3.29 (6.21)	−0.75 (2.82)	−2.00 (4.09)	−1.16 (4.17)
delta T + 4–T4	2.06 (3.40)	2.00 (4.36)	1.25 (2.49)	1.65 (2.94)	2.06 (3.82)

Delta T + 4 – T4: N = 18 in SARA < 20, N = 16 in Dose 400.

**Table 6:** Multiple linear models’ estimated differences (*p* values) in changes over time between severity or dose and the reference group.

	T4 vs. T0	T + 4 vs. T4
Peak VO_2_		
Severity (SARA 20–30)	1.347 (0.196)	0.212 (0.850)
Severity (SARA > 30)	0.715 (0.478)	1.242 (0.268)
Dose (400)	0.354 (0.661)	−0.205 (0.815)
SARA		
Severity (SARA 20–30)	0.282 (0.589)	−0.569 (0.234)
Severity (SARA > 30)	1.398 (0.010)	−1.552 (0.002)
Dose (400)	−0.304 (0.459)	0.637 (0.094)
Peak W		
Severity (SARA 20–30)	−1.485 (0.546)	2.290 (0.403)
Severity (SARA > 30)	0.962 (0.689)	−2.742 (0.315)
Dose (400)	0.189 (0.922)	−0.627 (0.770)
Septal wall thickness		
Severity (SARA 20–30)	0.335 (0.422)	−0.100 (0.769)
Severity (SARA > 30)	−0.220 (0.589)	−0.198 (0.559)
Dose (400)	0.147 (0.653)	0.441 (0.108)
Sokolow–Lyon index		
Severity (SARA 20–30)	−1.911 (0.317)	−0.034 (0.983)
Severity (SARA > 30)	0.444 (0.810)	−0.864 (0.588)
Dose (400)	0.651 (0.662)	0.408 (0.746)

Models are adjusted for age. Reference groups: SARA < 20 for severity, 200 mg/day for dose.

## Data Availability

The original contributions presented in the study are included in the article/Supplementary Material, further inquiries can be directed to the corresponding author.

## Supplementary Materials

The following supporting information can be downloaded at: https://www.mdpi.com/article/10.3390/children11080958/s1, Table S1: Overall and by dose average outcome measures scores.
