# Supplementary material for: A Pilot Phase 2 Randomized Trial to Evaluate the Safety and Potential Efficacy of Etravirine in Friedreich Ataxia Patients
Source: Children (Basel). 2024 Aug 9;11(8):958. doi: 10.3390/children11080958 (PMC11352957; doi:10.3390/children11080958)
Supplement: Supplementary file 1 [file children-11-00958-s001.zip › children-3115527-supplementary.pdf]

"A Pilot Phase 2 Randomized Trial to Evaluate the Safety and Potential Efficacy of Etravirine in Friedreich Ataxia Patients"

Table S1. Overall and by dose average outcome measures scores.

		**T–4**	**T0**	**T2**	**T4**	**T+4**	***p* value***
Peak VO_2		13.87 (3.08)	14.55 (3.36)		15.51 (3.20)	14.35 (2.88)	0.004
	dose 200	14.19 (2.75)	13.97 (3.26)		14.77 (3.29)	13.78 (2.95)	0.433
	dose 400	13.55 (3.44)	15.14 (3.45)		16.25 (3.02)	14.95 (2.77)	0.001
SARA		20.71 (8.19)	21.16 (8.52)	20.26 (8.83)	20.03 (9.05)	21.32 (8.52)	<0.001
	dose 200	20.03 (8.23)	20.38 (8.69)	19.65 (8.88)	19.41 (9.22)	20.35 (8.70)	0.003
	dose 400	21.38 (8.35)	21.94 (8.54)	20.88 (9.00)	20.65 (9.12)	22.34 (8.49)	<0.001
Peak W		20.44 (6.96)	20.29 (5.95)		23.24 (7.17)	22.12 (6.69)	0.007
	dose 200	21.24 (7.71)	19.29 (4.95)		22.00 (6.16)	21.29 (5.52)	0.152
	dose 400	19.65 (6.25)	21.29 (6.82)		24.47 (8.05)	23.00 (7.83)	0.013
Septal wall thickness		11.84 (1.94)	12.01 (1.92)		12.12 (2.05)	12.26 (1.93)	0.232
	dose 200	12.21 (2.09)	12.15 (2.12)		12.21 (2.13)	12.12 (1.97)	0.965
	dose 400	11.47 (1.76)	11.88 (1.75)		12.03 (2.03)	12.41 (1.93)	0.064
Sokolow-Lyon index		18.55 (9.66)	18.44 (7.93)		16.85 (7.51)	18.64 (8.04)	0.099
	dose 200	19.69 (11.29)	18.29 (8.26)		16.29 (7.56)	17.94 (8.00)	0.100
	dose 400	17.41 (7.90)	18.58 (7.83)		17.41 (7.64)	19.38 (8.28)	0.092

* P-values were obtained from One Way ANOVA.
